# Hypoxia-induced cancer cell reprogramming: a review on how cancer stem cells arise

**DOI:** 10.3389/fonc.2023.1227884

**Published:** 2023-08-08

**Authors:** Genevieve M. Abd, Madison C. Laird, Jennifer C. Ku, Yong Li

**Affiliations:** ^1^ Department of Orthopedic Surgery, Biomedical. Engineering, Western Michigan University Homer Stryker MD School of Medicine, Kalamazoo, MI, United States; ^2^ Medical Students, Western Michigan University Homer Stryker MD School of Medicine, Kalamazoo, MI, United States

**Keywords:** reprogramming, cancer stem cells, Yamanaka factors, metastasis, hypoxia

## Abstract

Cancer stem cells are a subset of cells within the tumor that possess the ability to self-renew as well as differentiate into different cancer cell lineages. The exact mechanisms by which cancer stem cells arise is still not completely understood. However, current research suggests that cancer stem cells may originate from normal stem cells that have undergone genetic mutations or epigenetic changes. A more recent discovery is the dedifferentiation of cancer cells to stem-like cells. These stem-like cells have been found to express and even upregulate induced pluripotent stem cell markers known as Yamanaka factors. Here we discuss developments in how cancer stem cells arise and consider how environmental factors, such as hypoxia, plays a key role in promoting the progression of cancer stem cells and metastasis. Understanding the mechanisms that give rise to these cells could have important implications for the development of new strategies in cancer treatments and therapies.

## Introduction

Cancer is one of the leading causes of death worldwide according to the latest data from the World Health Organization (WHO). In 2020, there were an estimated 9.9 million cancer-related deaths globally (1). The solid tumor environment, also known as the tumor microenvironment, refers to the surrounding cellular and non-cellular components that interact with and influence the behavior of solid tumors. It is a complex ecosystem that plays a crucial role in tumor growth, progression, and response to therapy. The solid tumor environment consists of various components, including cancer cells, stromal cells, immune cells, blood vessels, the extracellular matrix (ECM), and signaling molecules.

Solid tumors are composed of cancer cells that have undergone genetic alterations, enabling them to divide and grow uncontrollably. These cancer cells interact with other cells and components within the tumor environment. Additionally, stromal cells are also non-cancerous cells present in the tumor environment. This subset includes cancer-associated fibroblasts, which provide structural support to the tumor, produce extracellular matrix proteins, and secrete growth factors that can promote tumor growth (2). Other stromal cells, such as adipocytes and pericytes, may also be found in the tumor environment. The involvement of endothelial cells in cancer is primarily related to tumor angiogenesis, which is the formation of new blood vessels to supply nutrients and oxygen to the growing tumor.

The immune system plays a critical role in recognizing and eliminating cancer cells, thereby exerting a significant influence on the composition of the tumor environment. The involvement of endothelial cells in cancer is primarily related to tumor angiogenesis, whichis the formaAon of new blood vessels to supply nutrients and oxygen to the growing tumor. In response to the presence of cancer cells, immune cells, such as T cells, B cells, natural killer (NK) cells, dendritic cells, and macrophages, infiltrate the tumor microenvironment. This infiltration can be influenced by the chemotactic signals and interactions between cancer cells, stromal cells, and immune cells (3). Despite immune cell infiltration, tumors can create an immunosuppressive microenvironment. Tumor cells and stromal cells release factors, such as cytokines and chemokines, that inhibit immune cell activity and promote the recruitment of immunosuppressive cells. Regulatory T cells, myeloid-derived suppressor cells, and tumor-associated macrophages are examples of immune cells with suppressive functions that can be found in the tumor microenvironment.

The solid tumor microenvironment is also intrinsically shaped by the presence of vascularization, which is essential in providing the blood supply that is needed for the growth and survival of solid tumors. Blood vessels within the solid tumor environment deliver oxygen, nutrients, and growth factors to the tumor cells. The formation of new blood vessels, known as angiogenesis, is a crucial process in solid tumor development (4). However, the tumor vasculature can be abnormal, leading to inadequate blood flow and oxygenation in some regions of the tumor.

Additionally, the ECM significantly influences the solid tumor microenvironment. As a regulated mechanism comprising of network of proteins, carbohydrates, and other structural and support molecules, ECM remodeling in solid tumors has been demonstrated to exert profound effects on tumor behavior (5). The modified ECM can promote tumor cell migration, enhance tumor cell invasion into surrounding tissues, and facilitate metastasis. Furthermore, the tumor microenvironment contains various signaling molecules, including growth factors, cytokines, and chemokines, which further enhance tumor cell proliferation, migration, invasion, and angiogenesis.

Within the heterogenous tumor environment, a subset of cells, known as cancer stem cells (CSCs), are believed to play a key role in tumor growth, recurrence, and resistance to therapy. Studies have shown that the percentage of CSCs in a tumor can range from less than 1% to more than 50%, depending on the cancer type and stage (6). Based on decade-long studies, CSCs are thought to be especially important for the development and treatment of cancer clinically. However, it is unclear on the how, when, and where CSCs originate, and this remains a continuous research subject. One hypothesis is that the CSCs developed from cellular reprogramming, a process by which cells lose their original identity and acquire a different cell fate (7). In some cases, CSCs can also be differentiated into other cell types, which can contribute to tumor heterogeneity and tumor progression (7). Yamanaka factors can also induce the formation of induced pluripotent stem cells (iPSCs), which have some similarities to CSCs and can be used to reprogram CSCs (8). Several studies have shown that the overexpression of Yamanaka factors in cancer cells can induce a stem-like state, which is associated with increased tumorigenicity and drug resistance (8–10). This suggests that there may be a link between the reprogramming of cells to a pluripotent state and the acquisition of stem-like properties in cancer cells that increase their metastatic behavior.

Another important environmental factor, hypoxia, which refers to a deficiency in the amount of oxygen reaching the tissues, has been found to be related to the regulation of Yamanaka factors (11). Studies have shown that hypoxia can induce the expression of Yamanaka factors in cells, which can lead to the reprogramming of cells into a pluripotent state (11, 12). Hypoxia-inducible factor 1 (HIF-1) is a transcription factor that is activated under conditions of hypoxia, and it has been found to regulate the expression of Yamanaka factors in response to low oxygen levels (13). Furthermore, the activation of HIF-1 has been shown to be important for the survival and self-renewal of CSCs, which are thought to be responsible for tumor initiation, progression, and resistance to therapy (13). It has been suggested that the upregulation of Yamanaka factors in response to hypoxia may contribute to the maintenance of CSCs and the development of tumors.

Here, we first review different theories that illuminate the heterogeneity of how CSCs can arise and how a hypoxic environment can be involved in promoting CSCs. Secondly, we will review the commitment of Yamanaka factors as inducers of stem-cell like qualities seen in CSCs. Additionally, the unique interaction between CSCs and the extracellular matrix will be discussed in the context of CSC’s ability to significantly increase metastatic behavior. Lastly, we discuss how reprogramming can be used as a possible therapeutic strategy in CSC treatment.

## Cancer stem cells

“Omnis ellula e cellular (every cell stems from another cells).” This aphorism originated from Rudolf Virchow, a German physician whose publication made a major contribution to the cell theory in 1855. Virchow proposed that some tumors arise from embryonic-like cells with properties that are similar to stem cells (14). Over a century later, the existence of CSCs was identified in human acute myeloid leukemia (15). This was followed by the discovery of CSCs within a solid breast tumor environment (16). In 2006, Shinya Yamanaka, a stem cell researcher, discovered that adult somatic cells can undergo cellular reprogramming into an embryonic-like pluripotent state through transcription factors known collectively as Yamanaka factors (OCT3/4, SOX2, KLF4, and c-MYC) (17). Multiple studies have implicated that CSCs share critical properties with embryonic cells through the elevated expression of Yamanaka factors, which supports the impact of these pluripotency factors in tumorigenesis (18, 19). Considering these recent discoveries however, it is still not clear how CSCs arise in the tumor environment. It is important to note that the CSC population can change over time, as cells undergo genetic mutations or epigenetic changes that alter their properties. Additionally, the tumor microenvironment, which includes cells and molecules surrounding the tumor, can influence the behavior of CSCs and their ability to proliferate and differentiate.

## The different theories of how cancer stem cells arise

**Figure 1:**
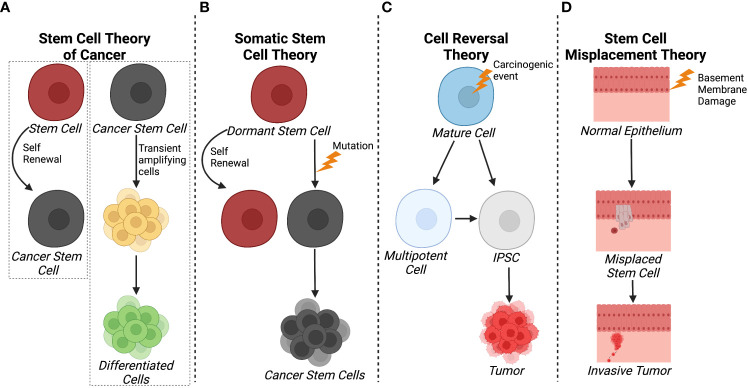
Schematics of different theories of CSC origin. **(A)** The stem cell theory of cancer postulates that cancer can arise from tissue stem cells and that CSC and/or cancer cells can give rise to normal, differentiated cells through transited amplifying cells. **(B)** In contrast, the somatic cell theory suggests that dormant stem cells become cancerous cells as a result of a mutation. **(C)** The cell reversal theory refers to a carcinogenic event that initiates cell de-differentiation to different epigenetic states that can develop into CSCs. **(D)** The stem cell misplacement theory alludes to basement membrane damage causing the misplacement of resident stem cells into the stoma leading to tumor formation and invasion. Created with BioRender.com.

The stem cell theory of cancer (**Figure 1A**) includes two major concepts: 1) cancer arises from stem cells that are present in the tissues of both children and adults; and 2) cancer cells originate from the cells of normal tissues, including stem cells, proliferating cells, and terminally differentiated cells (20). In each of the hypothesis on the origin of cancer, including field theory, chemical carcinogenesis, infection, mutation, and genetic change, stem cells are considered to be the core generator of cancer. According to the stem cell theory, cancer originates from the arrest in the maturation of stem cells. Additionally, CSCs and/or cancer cells can give rise and/or be composed of cells found in normal tissues including stem cells, transit amplifying cells, and terminally differentiated cells. Observations on the origins of teratocarcinomas and hepatocellular carcinomas led to another hypothesis where cancers arise due to arrest in stem cell maturation (21). Within the same decade, tumor transplantation studies supported the theory that cancer was maintained by a small population of stem-like cells (22, 23).

More currently, the somatic stem cell hypothesis (**Figure 1B**) suggests that mutation or chromosomal rearrangements in dormant stem cells could lead to CSC formation (24). Somatic stem cells have three stable features: 1) they generate identical cells and have long term self-renewal; 2) their progeny differentiates to functional, specialized cells; and 3) they faithfully respond to homeostatic controls within their environment (24). In contrast, CSCs undergo aberrant differentiation and do not respond to homeostatic controls as they undergo self-renewal (25).

With respect to carcinogenesis, the Cell Reversal Theory (**Figure 1C**) refers to a carcinogenic event on the cell and/or its environment that causes the cell to transition to a different epigenetic state, leading to abnormal proliferation in the absence of homeostatic controls (26). As a result, the cell enters into a deviant epigenetic program and becomes a CSC according to environmental influences and its stage of development (26). Given that there is a plethora of epigenetic states, it is more probable that a small fraction of these transitioning cells survives and selects an evolutionary advantageous route, the stem cell or pluripotent route, rather than waiting on the correct and successive environmental inputs that lead to enhanced proliferation, therapy resistance, and invasion (26).

Another hypothesis relating to breast cancer pathology is the stem cell misplacement theory (**Figure 1D**), which states that misplaced epithelial stem cells arrive in the wrong connective tissue stroma which leads to invasive cancer, known as an *in situ* carcinoma (27). Thus, the seed of the cancer, which is a misplaced, normal epithelial stem cell, can migrate to basement membrane due to leakage or trauma. This epithelial stem cell could be identified as a foreign invader by the immune system or it could escape the immune system, ensuring its survival without triggering an immune response. Environmental factors within the basement membrane, including inflammation, trauma, aging, and degeneration, create favorable conditions for the mislocalization of normal epithelial stem, thereby increasing the probability of stem cell arrest and development of invasive cancer. This hypothesis is based on evidence that ductal carcinoma *in situ* has a higher frequency of HER2 gene amplification/overexpression (50-60%) compared to invasive breast cancer (20-30%) (27). However, it was noted that when the invasive breast cancer was mixed with ductal carcinoma in situ, the invasive cancer cells were HER2-negative while the ductal carcinoma *in situ* was HER2-positive. This indicated that HER2 amplification/overexpression had no advantage compared to HER2-negative in developing into an invasive cancer type (27). Overall, most theories agree that CSCs emerge as a result of accumulated epigenetic and/or genetic alterations arising from normal stem cells or cancer cells while fewer evidence suggest a “wrong place, wrong time” idiom scenario in *in situ* carcinoma.

## Cancer stem cell origin

Which arose first, the cancer cell or the CSC? To illuminate this question, tumor cells can arise either from transformed differentiated cells or transformed resident stem cells (28, 29). Transformation is likely to arise from environmental inputs such as tissue regeneration (30), infections (31), toxins (32), as well as intrinsic and non-intrinsic factors that lead to DNA mutations (33). As the transformation process progresses in differentiated cells, oncogenes are overexpressed while tumor suppresser genes are deactivated leading to the de-differentiation of cells, uncontrolled proliferation, and the acquisition of stem-like characteristics (26).

Tumors that originate from CSCs are thought to follow a unidirectional hierarchy, meaning that the CSC population initiates tumor growth (29). Thus, CSCs divide asymmetrically to maintain a subpopulation CSCs while also generating transient cells that undergo symmetric divisions with highly proliferative capabilities (34). Current data on solid and hematological cancers has suggested three models that are not mutually elusive to CSCs: 1) the hierarchical model in which cell of the stem/progenitor hierarchy are susceptible to transformation (15); 2) the CSC model in which a small subpopulation of cells promotes tumor initiation and growth (35); 3) the clonal evolution model which states that genetic instability from genetic alteration worsen over time leading to increased tumor aggressiveness, resistance, and heterogeneity (36). The validity of all three models in explaining the origin of CSCs attributes to the cellular plasticity seen with different cancer types (37).

The stem cell theory of cancer, somatic stem cell theory, cell reversal theory, and stem cell misplacement theory are all concepts based on the microenvironment of solid tumors. In contrast, leukemias, liquid tumors of the blood, represent a unique and contrasting environment for the origin or development of CSCs. Up until 1997, little was known of the origin of the target cell in the hematopoietic stem cell hierarchy for leukemic transformation (15). Two landmark studies involving immune-deficient mice models showed that leukemic cells expressing the same markers seen in adult human hematopoietic stem cells (CD34+CD38−) initiated hematopoietic malignancy (15, 38). These leukemia-initiating cells were termed leukemia stem cells or CSCs. To date, leukemia stem cells have been identified in both acute myeloid leukemia and acute lymphoblastic leukemia (39). These cells share characteristics with normal hematopoietic stem cells, including the ability to self-renew and generate differentiated progeny. CSCs in leukemias are believed to be responsible for the uncontrolled proliferation of leukemic cells and the ability of the disease to recur after treatment. Additionally, CSCs in leukemias are often characterized by the expression of specific cell surface markers, such as CD34, CD38, and CD123, among others, which can be isolated to study CSC populations in leukemia samples.

The exact origins of leukemia stem cells remain a subject of ongoing research and investigation. Leukemia is a complex disease that arises from genetic mutations and abnormalities in blood-forming stem cells. While the specific mechanisms underlying the development of LSCs are not fully understood, a couple of theories have been proposed based on scientific evidence and observations. One prevailing theory suggests that leukemia stem cells originate from hematopoietic stem cells or progenitor cells that acquire somatic mutations in critical genes responsible for regulating normal cell growth and differentiation (40). These mutations can lead to uncontrolled proliferation and the development of leukemia. Another theory suggests that leukemia stem cells arise from clonal evolution (41). Leukemia is characterized by genetic heterogeneity, indicating that leukemia stem cells and their progeny undergo clonal evolution over time. This means that as the disease progresses, subclones with distinct genetic mutations may emerge, leading to treatment resistance and disease relapse.

## Effects of hypoxic tumor environment on cancer stem cells

Worldwide, solid tumors account for the highest levels of morbidity and mortality and are the most common form of cancer (42). A hallmark of the tumor environment is hypoxia, especially in rapidly growing solid tumors where the oxygen levels can range from 0% to 2% compared to normal physiological levels of 4% to 9% oxygen (43). These low oxygen levels have been noted in prostate (44), cervix (45), breast (46), head and neck cancers (47), with levels depending on the size, stage, initial oxygenation level, and the method applied to measure the oxygen within the solid tumor (48).

**Figure 2:**
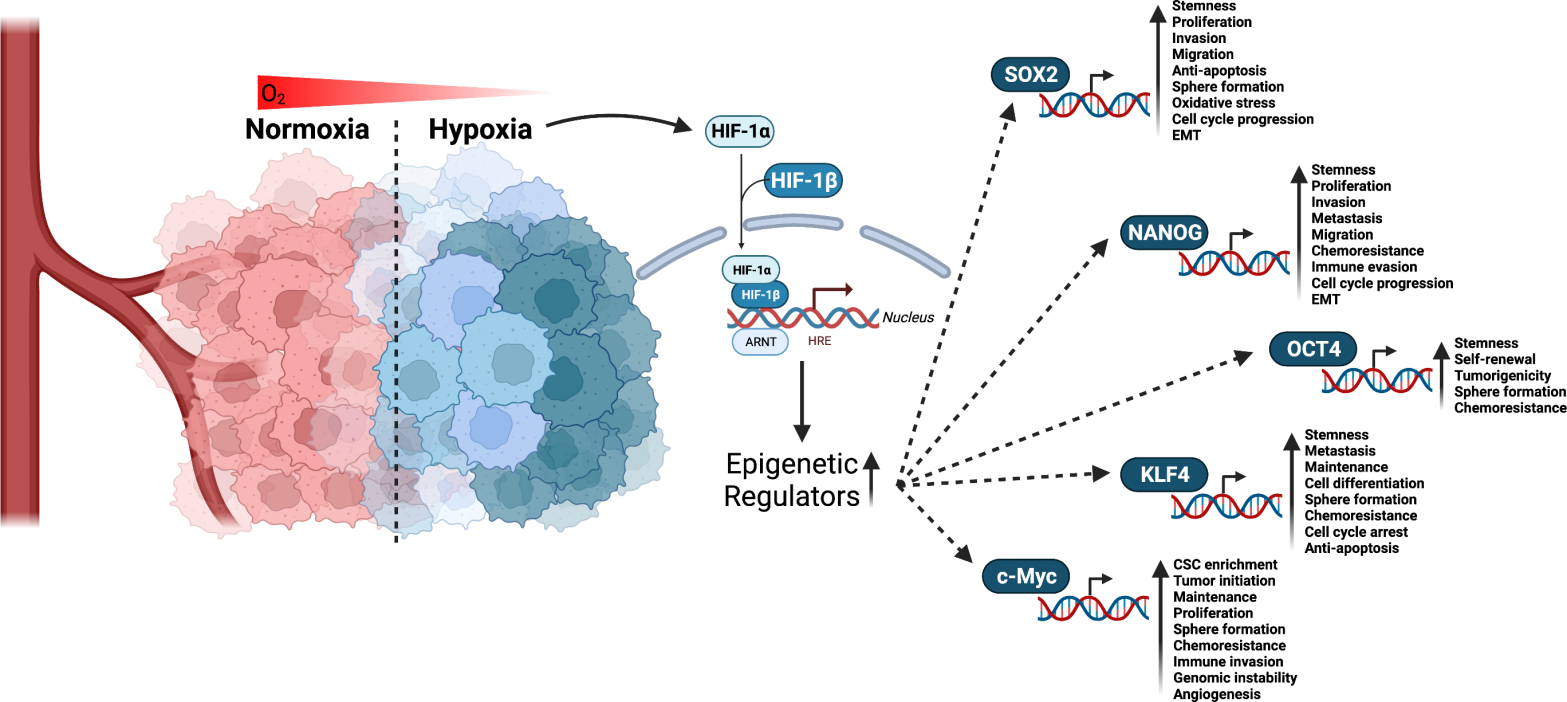
Interaction of HIF factors and stemness generation through pluripotent stem cells markers. Hypoxia is a known hallmark of solid tumors and occurs when cells are deprived of oxygen. This causes the cells to switch to alternative metabolic pathways that allow them to survive and continue to grow. As a result, adaptations to hypoxia can lead to changes in the way tumor cells behave, making them more aggressive and resistant to chemotherapy and radiation. Given that tumors have a high metabolic demand and often outgrow their blood supply, HIFs are activated to regulate the expression of numerous genes that promote cell survival, proliferation, angiogenesis, and resistance to therapy. HIF-1α and HIF-2α have been linked to promoting CSCs stemness and phenotype through the upregulation of epigenetic regulators including SOX2, NANOG, OCT4, KLF4, and c-Myc. Created with BioRender.com.

A core mechanism of stemness generation and maintenance induced by hypoxia are hypoxia inducible factors (HIFs) (**Figure 2**) (49, 50). HIFs are a helix-loop-helix-Per-ARNT-Sim (bHLH-PAS) containing transcription factor (51). HIF-1 is a heterodimer made up of α and β subunits. These heterodimers translocate in the nucleus and interact with specific DNA sequences, called HIF-responsive elements, leading to activation or repression of gene expression. So far, three different genes are known to encode a subunit of HIF: HIF1α, HIF2α, and HIF3α. All three heterodimerize with the HIF-1β subunit and are subject to posttranslational regulation that is dependent on oxygen levels in the environment (52). In most cases, HIF3α is thought to be a negative regulator of HIF1α and HIF2α. However, a study using zebrafish showed that HIF3α is degraded under normoxia (21% O_2_) and when overexpressed under hypoxia binds to target gene promoters to upregulate expression (53). HIF1α and HIF2α expression is specific to tissue type and the time course of induction by hypoxia (54). For example, HIF-1α expression levels peak early under hypoxia while HIF-2α expression slowly rises and is more sustained (54). With respect to CSCs, a study investigating glioblastoma stem cells demonstrated differential expression of HIF-1α and HIF-2α in stem and non-stem cancer cell populations. HIF-2α was found to be present within the CSC population, while HIF-1α was present in both stem and non-stem cancer populations. In the same study, knockdown of both HIF-1α and HIF-2α attenuated stem cell and non-stem cell survival and proliferation. HIF-2α knockdown led to growth restriction for stem cells, while HIF-1α knockdown resulted in reduced growth in stem and non-stem cells. HIF-1α and HIF-2α were also shown to be required for VEGF expression in stem cells, but only HIF-1α expression was required for VEGF expression in non-stem populations (55). While HIF-1α has been shown to mediate angiogenesis (56), metabolic reprogramming (57), invasion (58), metastasis (58), and epithelial-mesenchymal transitioning (59), this study suggests HIF-2α serves a unique role in CSC development and maintenance. It is important for promoting the CSC phenotype (49), prompting de-differentiation of cancer cells to CSCs (60), as well as inducing the expression of pluripotent stem cell markers, OCT4, NANOG, SOX2, KLF4, c-MYC (**Figure 2**) (61).

## Interaction of hypoxia and Yamanaka factors in cellular reprogramming

**Figure 3:**
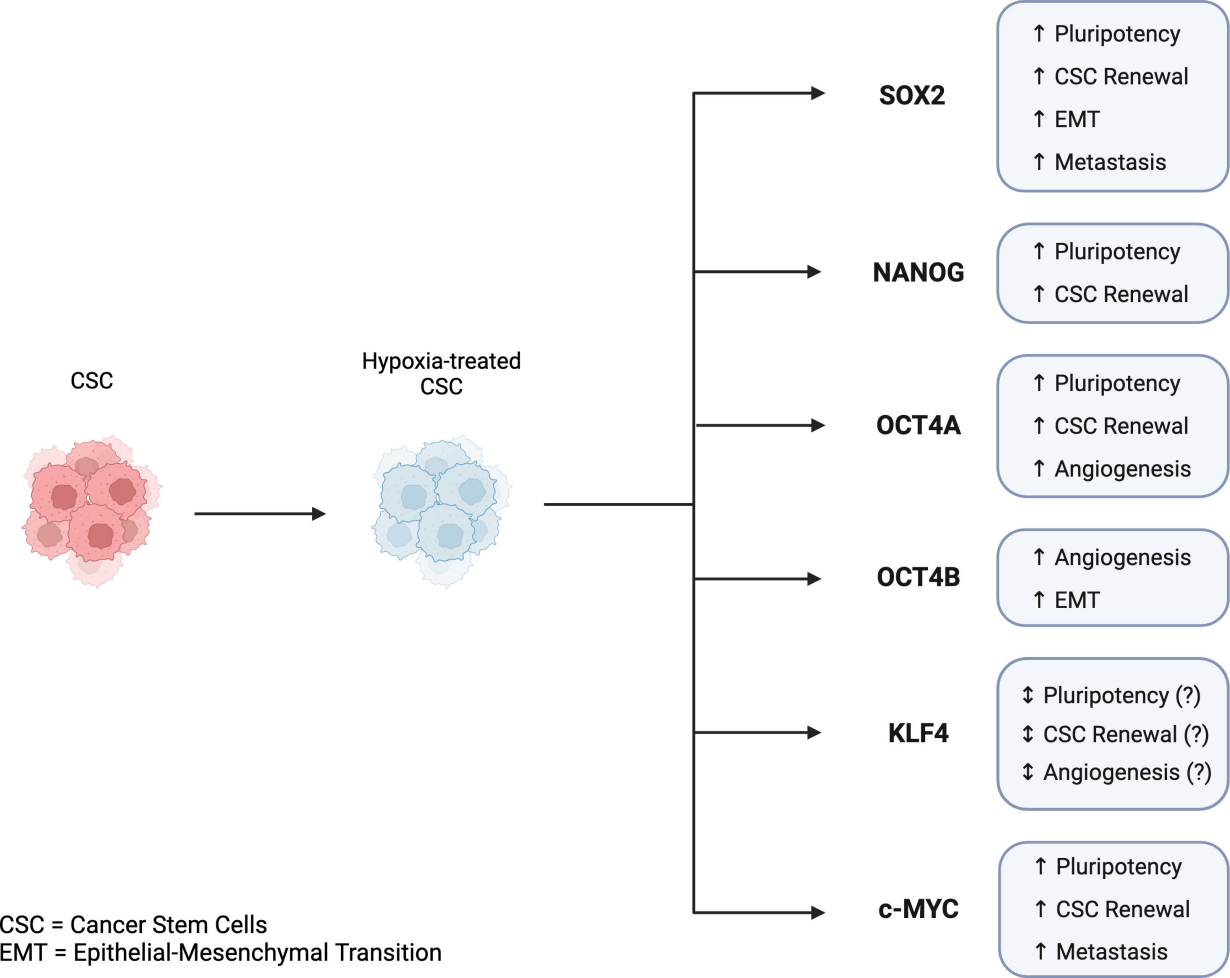
Key features of Yamanaka transcription factors in cancer stem cells and tumor progression in hypoxic conditions. Hypoxia-induced expression of Yamanaka transcription factors is associated with CSC reprogramming, CSC maintenance, and tumor progression. SOX2, NANOG, OCT4A, KLF4, and c-MYC are all linked to pluripotency, CSC renewal, angiogenesis, EMT, and metastasis. While KLF4 has been shown to play a role in CSC reprogramming, its mechanism and regulation in the reprogramming of CSCs is not fully understood. Created with BioRender.com.

CSCs are classified by expression of stemness-related markers. Several markers have been reported to be expressed in CSCs including the Yamanaka transcription factors OCT4, NANOG, SOX2, KLF4, and c-MYC (62, 63). Discovered by Dr. Shinya Yamanaka in 2006, these transcription factors can collectively reprogram adult cells into induced pluripotent stem cells (iPSCs) and are considered CSCs biomarkers. Studies have also suggested that expression levels of Yamanaka factors are associated with the prognosis of several cancer types, and subsequently can be useful in assessing patient diagnosis and treatment decisions (17, 63). As previously discussed, hypoxia and Yamanaka factors have been found to be related in the sense that hypoxia can induce the expression of Yamanaka factors, which may contribute to the reprogramming and maintenance of cells into a pluripotent state (64) (**Figure 3**).

### SOX2

SOX2 is a transcription factor expressed early on in embryonic development and throughout adulthood. It functions as part of the core transcriptional network to maintain cell pluripotency and self-renewal, in addition to regulating various other cell functions such as metabolism, inflammation, and development (65–67) In the cell, SOX2 binds directly to DNA targets to maintain expression of pluripotency-associated genes and inhibit expression of genes associated with differentiation. Furthermore, elevated levels of SOX2 expression have also been associated with several cancers including breast (68), prostate (69), and pancreatic cancer (70).

Specifically in breast cancer, SOX2 has been shown to be involved in the development of breast CSCs (BCSC) (71, 72). In addition, a recent study demonstrated that in hypoxic conditions, SOX2 expression increases in a time-dependent manner facilitating hypoxia-induced breast cancer cell migration *via* expression of NEDD9, which further induced Rac1 and HIF-1α expression (68). A different study found that in esophageal squamous cancer cells, SOX2 promotes the expression of SLUG, a key regulator in the hypoxia-induced epithelial to mesenchymal transition, through activation of STAT3 HIF-α signaling (73, 74). In addition, a study on prostate cancer found hypoxic-induced SOX2 and HIF-α expression promoted cell invasion and sphere formation, and therefore facilitated metastasis (75). Overall, SOX2 appears to play a complex role in hypoxia, facilitating both the expression of HIF-1α and its downstream pathways, and promoting tumor progression and invasion.

### NANOG

Abnormal expression of NANOG, a differentiated HOX domain protein, has been reported in several human cancers including breast cancer (76), lung cancer (77), adenocarcinoma (78), and gastric cancer (79). Although the exact molecular mechanisms of NANOG remains poorly understood, NANOG expression has been associated with both the development and maintenance of pluripotent state of CSCs (80). For example, in a pancreatic cancer tissue microarray analysis of 43 cases, high expression of NANOG predicted worse prognosis, and knockdown of both NANOG and OCT4 inhibited stemness of pancreatic cancer cells (81). Numerous studies have also identified dynamic roles of NANOG in the hypoxic tumor microenvironment including but not limited to the activation of tumor autophagy in non–small lung carcinoma cells (82), colony formation in colorectal CSCs (83), and resistance to mediated lysis in non-small lung cell carcinoma cells (84).

Recent studies have also identified that specifically in BCSC, HIF-1 and NANOG cooperatively mediate stem cell maintenance in hypoxic conditions. HIF-1α has been demonstrated to be required for NANOG function in stem-cell maintenance by activating transcription of the ALKBH5 gene, which stabilizes NANOG mRNA through demethylation. NANOG then serves as a HIF-1 coactivator for the transcription of telomerase reverse transcriptase, an essential component for telomerase activity in telomere elongation (85, 86). Additionally, another study elucidated a potential role of hypoxia-induced NANOG in tumor immunosuppression in melanoma *via* enhancement of TGF-β1 expression (87). Overall, these studies suggest a cooperative relationship of HIF-1 and NANOG in hypoxic conditions and stem cell maintenance, while NANOG’s dynamic roles continue to be elucidated.

### OCT4

OCT4, a member of the Pit-Oct-Unc (POU) transcription factor family, plays a critical role in self-renewal, pluripotency, and maintenance of CSCs in several cancer types (88). OCT4 protein has three isoforms (OCT4A, OCT4B, and OCT4B1), with OCT4A often referred to as simply OCT4. OCT4 (OCT4A) is recognized as one the most important transcription factors in cancer. Elevated expression levels have been found in several cancers including lung, breast (89), bladder (90), liver (91), and pancreatic cancer (79).

Specifically, in germ-line tumors, knockdown of OCT4 was demonstrated to directly reduce cell proliferation and stemness (92). In non-small cell lung cancer cells, a recent study found that both HIF-1α and HIF-2α upregulate SOX2 and OCT4 expression, which collectively regulate CSC formation through upregulation of CD133 and CD44 stem cell markers (93). However, in a separate study, HIF-2α and not HIF-1α was shown to be critical for expression of OCT4, specifically OCT4B, in lung cancer cells. OCT4B expression was further identified to promote cancer invasion through enhancing SLUG expression in a similar mechanism to SOX2 (94). Both OCT4A and OCT4B have also been shown to have pro-angiogenic characteristics, facilitating the transition of CSC to tumor endothelial-like cells in liver cancer (95). Overall, while it appears the connection of OCT4 and hypoxia has been established in literature, the exact mechanism of expression and function of OCT4 and its isoforms requires further research.

### KLF4

KLF4 is a bifunctional zinc-finger transcriptional factor that is widely expressed in many tissues. It plays a role in several physiological processes including cell differentiation, inflammation, apoptosis, inflammation, and angiogenesis (96). In cancer, KLF4 has been demonstrated to be either oncogenic or anti-oncogenic depending on the cancer type, and therefore performs unique functions in different CSCs. For example, in colon cancer KLF4 is a potent tumor suppressor, while in melanoma KLF4 expression promotes cell proliferation (97, 98).

Studies have shown that HIF-1 directly enhances KLF4 gene expression under hypoxic conditions, and KLF4 plays a critical role in regulating cell response to hypoxia (99, 100). KLF4 has been shown to inhibit the expression of HIF-1α, the regulatory subunit of HIF-1, under normoxic conditions (21% O_2_), thereby reducing the cellular response to hypoxia (101). In addition, KLF4 has been implicated in regulating hypoxia-induced apoptosis and angiogenesis (102). Specifically in certain cancer cells, KLF4 has been demonstrated to promote hypoxia-induced apoptosis by upregulating the expression of pro-apoptotic genes, and to inhibit hypoxia-induced angiogenesis by downregulating the expression of pro-angiogenic factors such as VEGF (vascular endothelial growth factor) (103). Overall, the connection between KLF4 and hypoxia appears to be complex, with KLF4 playing a role in regulating the cellular response to hypoxia and hypoxia in turn inducing KLF4 expression.

### c-MYC

c-MYC is an essential transcription factor part of the basic helix-loop-helix (bHLH) DNA-binding proteins involved in regulating normal cell processes including cell division, metabolism, differentiation, cell death, and maintenance of stem cells properties. Considering its myriad of functions, c-MYC is tightly regulated by developmental and mitogenic signals in normal cells, and dysregulation has been linked to oncogenic potential in several cancers. Studies have found that sustained c-MYC overexpression leads to oncogenic and epigenetic reprogramming *via* downregulation of lineage-specific transcription factors. This de-differentiation creates a more progenitor-like state that allows for acquisition of stem cell traits by c-MYC activation of *de novo* oncogenic enhancers, supporting CSC development (104).

Studies have suggested a complex interplay between HIF and c-MYC under hypoxic conditions. HIF-1α has been shown to inhibit c-MYC activity, while HIF-2α activates c-MYC activity *via* stabilization of c-MYCs transcriptional activation and repression functions. C-MYC also directly promotes HIF-2α expression, while its overexpression overall increases HIF levels and activity. Therefore, in cancer, when c-MYC levels are high, HIF and c-MYC collectively remodel cellular processes to increase oncogenic potential including angiogenesis and metastasis (105). A recent study on breast cancer cells adds to this complexity. Recently, Zhu et al. identified a hypoxia-induced long non-coding RNA (lncRNAs) that contributes to the development of BCSC by creating a complex with insulin-like growth factor 2 mRNA-binding protein 1 (IGF2BP1) that stabilizes c-MYC mRNA (106). Overall, the relationship between c-MYC and hypoxia seems to be well established, but the exact modulators of c-MYC stabilization and expression continue to be investigated.

## The role of cancer stem cells in metastasis

Metastatic disease remains the primary cause of cancer mortality. Despite recent advancements in cancer therapies, survival in metastatic setting is still poor. 90% of cancer-related deaths are due to metastatic disease rather than primary tumors. Historically, cancer progression has been firmly established in mutational processes and clonal expansion (107). However, recent studies have implicated CSCs in the resistance (108) and recurrence of tumors (109). Studies have also linked direct reprogramming of somatic cells to cancer metastasis (110). Similarities between somatic cells and CSCs suggest an association between reprogramming in CSCs and progression of disease (111).This relationship could be the basis for metastatic disease, driving tumor initiation and growth. However, the underlying mechanisms of CSC reprogramming and tumor progression remain unclear.

The epithelial-mesenchymal transition (EMT) is a developmental process that leads to the transformation of epithelial cells into mesenchymal cells. While EMT is accepted to occur during embryogenesis, it has also been recognized in CSCs (112). In 2005, the “migratory cancer stem (MCS) cell” theory established the association between CSCs and EMT, which describes a ‘mobile CSC’ as a stationary CSC with partial EMT that disseminates and retains stem cell features, forming metastatic colonies (113). The acquisition of migratory qualities through EMT could then be a critical feature of CSCs in the growth, invasion, and dissemination of tumors, leading to the poor prognosis seen in metastasis. However, it is unclear whether only CSCs are responsible for the initiation of metastatic growth, or if there are other factors driving CSCs for pre-metastatic development.

Another concept that implicates CSCs in the progression of metastatic disease is their influence on the microenvironment. It is well known that hypoxia promotes tumor changes, such as EMT and dysfunctional vascularization, leading to cell migration and metastatic disease (114). Hypoxia has also been shown to stimulate CSCs. The increase in reactive oxygen species due to hypoxia promotes EMT in CSCs, resulting in the production of VEGF (115). While VEGF is fully established as an angiogenic factor, it has also been shown to drive malignancy through other mechanisms. For example, VEGF promotes breast and lung CSC renewal *via* VEGF receptor-2/STAT3-mediated upregulation of c-MYC and SOX2 (116). However, variable VEGF expression among cancer subtypes suggests involvement of other potential factors in the microenvironment.

**Figure 4:**
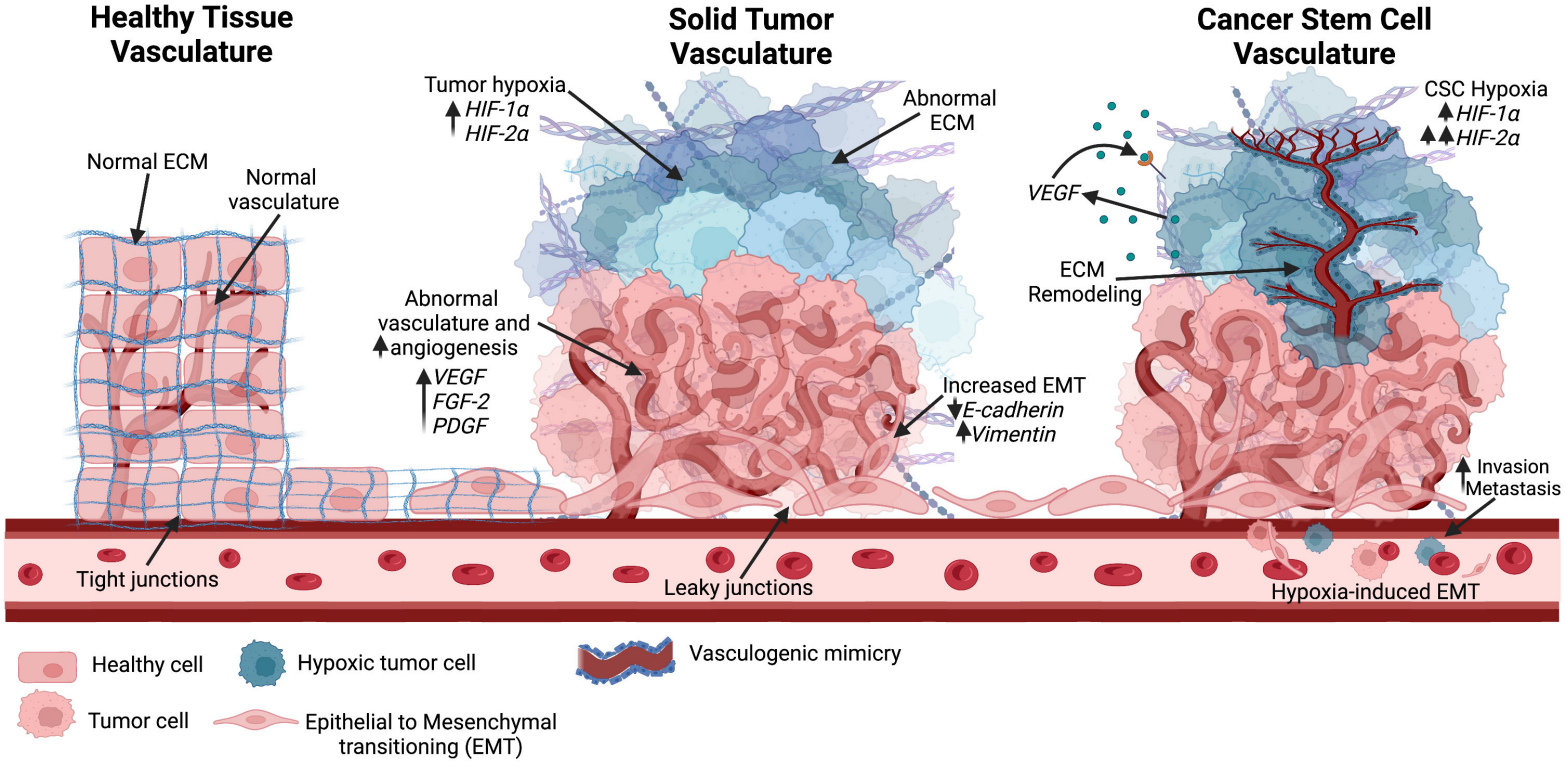
Different vascular structures seen in the tumor environment. Healthy tissue consists of normal vascularization, ECM, and tight junctions. As the solid tumor progresses, rapid tumor growth increases hypoxic niches and leads to abnormal angiogenesis. Abnormal vascular formation is also seen, allowing for metastasis through enhanced EMT. Hypoxia is known to increase HIF factors. As the hypoxic tumor environment progresses, HIF-2α expression rises and further promotes stemness in the hypoxic environment. Cancer stem cells have been positively correlated with vascular mimicry, in which ECM remodeling occurs along with epithelial-endothelial transitioning to create new vasculature into the hypoxic environment. Created with BioRender.com.

For decades, tumor vascularization *via* angiogenesis was considered the primary method of blood supply for tumor growth (117). In 1999, Maniotis et al. reported highly invasive and metastatic human melanoma cells with tumor growth independent of angiogenesis, suggesting the generation of vascular channels by cancer cells without the involvement of endothelial cells (118). This process was termed vascular mimicry (VM) (**Figure 4**). Tumors with VM lead to a worse prognosis, and evidence has indicated that VM may be used as an independent prognostic factor for survival (119). However, the underlying mechanisms of the initiation of VM are still not well understood. Recently, evidence has suggested an association between VM and CSCs. CSCs were found to be positively correlated to triple negative breast cancer subtype and VM in human invasive breast cancer (120). Another study found that a hypoxic tumor microenvironment increased the population of CSCs, thereby accelerating VM channel formation in triple negative breast cancer (121). These findings could explain the invasive nature of treatment-resistant tumors.

## Therapeutic approaches in treating cancer stem cells

Many strategies have been implemented for cancer treatment including surgery, radiotherapy, chemotherapy, and targeted therapies. These strategies have been essential in reducing cancer death rate by 1.5% from 2019 to 2020 (122). Furthermore, an overall cancer death reduction of 33% has been reported since 1991 with mortality declines seen in leukemia, melanoma, kidney, and lung cancers (122). However, traditional cancer treatments are not always effective, especially with malignant tumors (123). A few reasons for unsuccessful treatment outcomes are metastasis, recurrence, cancer heterogeneity, resistance to the treatment, and immunological escape [L. Yang et al., (79)]. All of these factors can be attributed to the characteristics seen in CSCs present in the tumor environment. Therefore, CSCs are considered promising targets for cancer treatment.

Tumor hypoxia is considered one of the most inimical factors that leads to treatment resistance (124). Radiotherapy generates reactive oxygen species, causing irreversible cellular DNA damage and apoptosis (125). Currently, radiotherapy is most often used to treat head, neck, breast, cervix, prostate, and eye cancers. However, cancer cells experiencing hypoxia are likely to be three-times more resistant to radiation (126). On the other hand, CSCs are considered to be the “seeds” of cancer and are believed to be “awakened” with radiation, leading to recurrence and metastasis post-radiation (88). As for chemotherapy, oxygenated cells are essential for successful drug delivery (127). Thus, hypoxic cells, including CSCs, escape chemotherapy due to alterations in apoptotic pathways and DNA damage repair systems (128). Additionally, the restricted/abnormal vasculature of the hypoxic tumor environment contributes to inadequate transport of anti-tumor drugs into the tumor tissue (129). Immunotherapy is one of the more recent developments in cancer therapies (130). However, there is strong evidence that hypoxia suppresses T cells and support tumor associated macrophage polarization (131).

In the past few years, multiple efforts have been made to directly target CSC including biomarker-mediated targeting (79), targeting of mitochondria (132), targeting of CSC related genes (133), and targeting of epigenetics associated with CSCs (134). For example, OCT4 and LIN28, pluripotency factors are highly expressed in embryonic stem cells, were also found to be co-expressed in a sub-population of epithelial ovarian cancer cells (135). It was demonstrated that targeting these two pluripotent factors with RNA interference significantly reduced cell growth and survival of epithelial ovarian cancer cells suggesting that OCT4 and LIN28 were beneficial targets for in epithelial ovarian cancer patients. Further relating to OCT4 targeting, an oncolytic adenovirus was developed to retain its antitumor activity in a hypoxic environment (136). This OCT4-dependent oncolytic adenovirus was driven by nine copies of OCT4 response element as were a hypoxia response element to target bladder cancer cells that overexpressed HIF-2α and OCT4, which include bladder CSCs.

While it is recognized that benign cells can become CSCs, the real question is whether CSCs can be genetically and epigenetically reversed back to a benign phenotype. Thus, rather than targeting overexpressed pluripotent factors as seen before, a pioneer strategy would involve transcription factor-mediated cancer cell reprogramming (17). Currently, cancer reprogramming has been met with some success. For example, epigenetic modifications in melanoma cells were induced by reprogramming them into induced pluripotent cancer cells that were able to differentiate into non-tumorigenic lineages (65). In the direction of immunotherapy, cellular reprogramming has been used to generate T-cells and natural killer cells with enhanced anti-tumor properties (137). Thus, patient-tailored immune cell types, including macrophages and dendritic cells, can also be generated *via* reprogramming of lineage-specific master regulators that can induce unique cell identities, *in vivo*. The involvement of cancer reprogramming has been taken a step further in the direction of therapeutic cancer vaccination. In a recent study by Majeti et al. (2023), myeloid lineage reprogramming was used to convert murine leukemia cancer cells into tumor reprogrammed-antigen presenting cells (138). These reprogrammed tumor cells demonstrated a myeloid phenotype and function as well as the ability to process and present endogenous tumor associated antigens that could be used to elicit cancer specific responses. With regards to human cancer cell lines, that is brain, uterine cervix, lung, colon, bladder, and synovium derived cell lines, double-network hydrogel has been utilized to create spheroids with elevated levels of stemness-related genes SOX2, OCT3/4, and NANOG *in vitro* within 24h of seeding (139). The results seen in the cells lines were compared to a highly malignant human brain cancer glioblastoma to confirm that the findings of elevated CSCs markers were not cell-line specific. Thus, spheroid formation highly mimics the CSC niche in the tumor environment and offers a method of *in vitro* reprogramming to identify reagents that can be used to specifically investigate CSCs.

On the flip side to successful reprogramming efforts, some cancer types resist or have limited reprogramming abilities. For example, human benign and malignant MCF10A and MCF7 breast cancer cell lines showed resistance to an induced reprogramming using retroviral reprogramming method (140). This study found that there were 29 candidate barrier genes with RNA-sequencing that could explain the roadblock seen in cancer cell reprogramming in these breast cancer cell lines. It has been noted that cancer cell reprogramming is more difficult and not well understood compared to somatic cells (141). One reason is that features that are observed in pluripotency, including morphology, specific gene expression, clonal expansion, immunocytochemistry, and teratoma formation are not well characterized, especially in cancer stem characterization (140). Another reason is that the differences between malignant and benign cancer cells have still not been fully characterized and require much more exploration.

## Conclusion

The origin of CSCs is complex and multifactorial, involving a combination of genetic and epigenetic changes, as well as changes in the cellular microenvironment. Hypoxia is a critical factor in the development and maintenance of CSCs. Hypoxia induces the activation of various signaling pathways that promote the survival and self-renewal of CSCs, leading to increased tumor growth and resistance to therapy. The adaptation of cancer cells to a hypoxic environment involves the upregulation of HIFs, which play a crucial role in the maintenance of CSCs. HIFs induce the expression of genes involved in cell survival, angiogenesis, and metabolism, providing a selective advantage to CSCs in hypoxic tumors. In addition, hypoxia also induces epigenetic changes that alter gene expression and contribute to the maintenance of CSCs. Yamanaka factors have been suggested as potential CSC markers since Yamanaka factors can induce a stem-like state in cancer cells, leading to the acquisition of cancer stem cell properties. The connection between Yamanaka factors and cancer stem cells lies in the similarities observed between induced pluripotent stem cells and cancer stem cells. Induced pluripotent stem cells generated by the expression of Yamanaka factors share certain characteristics with CSCs, such as self-renewal ability and the potential to differentiate into different cell types. This suggests that there may be overlapping mechanisms or molecular pathways involved in the generation and maintenance of both induced pluripotent stem cells and CSCs. Moreover, studies have indicated that the overexpression of Yamanaka factors can induce a stem-like state in cancer cells, leading to the acquisition of CSC-like properties. This includes enhanced self-renewal capacity, increased tumorigenicity, resistance to therapy, and the ability to initiate new tumors when transplanted into animal models. Additionally, emerging evidence suggests that hypoxia may influence the expression and activity of Yamanaka factors in cancer cells. Hypoxia has been shown to upregulate the expression of Oct4, Sox2, and c-Myc, among other pluripotency-related genes, potentially inducing a stem-like state. This connection raises the possibility that hypoxia could contribute to the acquisition or maintenance of CSC properties through the modulation of Yamanaka factors. By targeting and studying these markers, scientists can gain insights into the mechanisms underlying cancer initiation, progression, and treatment resistance. Understanding the mechanisms by which hypoxia regulates CSCs is crucial for developing effective therapies that target these cells and to prevent cancer recurrence. Further research is needed to fully elucidate the complex interplay between hypoxia, CSCs, and Yamanaka factor expression to identify new therapeutic targets that can be exploited for the treatment of cancer.

## Future directions

Advancements in CSC research have highlighted their critical role in tumor initiation, progression, metastasis, and therapeutic resistance. As we look ahead, several promising directions emerge that offer potential breakthroughs in understanding and targeting CSCs, particularly in the context of hypoxia and Yamanaka factors.

Firstly, investigating the interplay between CSCs and the hypoxic tumor microenvironment presents a compelling avenue for research. Hypoxia, or low oxygen levels, is a common feature of solid tumors and has been shown to promote CSC maintenance and metastasis. Future studies could focus on unraveling the molecular mechanisms underlying CSC adaptation to hypoxia, which may involve the activation of specific signaling pathways and epigenetic modifications. Understanding how hypoxia influences CSC properties, such as self-renewal and differentiation, will be crucial in developing novel therapeutic strategies to target these resilient cells within the tumor niche.

Secondly, exploring the role of Yamanaka factors in cancer stemness offers exciting prospects. The Yamanaka factors, initially identified for their ability to induce pluripotency in somatic cells, have been found to reprogram differentiated cancer cells back into a stem-like state. Investigating the precise mechanisms by which Yamanaka factors modulate CSC properties could pave the way for innovative approaches in cancer therapy. Utilizing these factors to induce differentiation in CSCs or, conversely, to reprogram them into less aggressive cell types could hold great therapeutic potential, potentially disrupting tumor growth and reducing resistance to conventional treatments.

Moreover, combinatorial approaches that integrate hypoxia-targeting strategies with Yamanaka factor-based therapies may yield synergistic effects in eradicating CSC populations. By targeting CSCs both within the hypoxic tumor microenvironment and at the molecular level through Yamanaka factors, researchers may enhance treatment efficacy and limit disease recurrence.

Lastly, translating these research findings into clinical applications remains a priority for future investigations. Developing therapeutics that specifically target CSCs, while sparing normal stem cells, poses a significant challenge. Nevertheless, progress in identifying unique CSC markers and understanding the signaling pathways that regulate CSC self-renewal provides promising opportunities for developing targeted therapies. Moreover, exploring innovative drug delivery systems that can effectively penetrate the hypoxic tumor regions may improve treatment outcomes.

In conclusion, the study of cancer stem cells, hypoxia, and Yamanaka factors represents a cutting-edge frontier in cancer research. Advancing our understanding of the molecular mechanisms governing CSC behavior within the hypoxic microenvironment and harnessing the potential of Yamanaka factors to reprogram these cells hold great promise for revolutionizing cancer treatment strategies. As interdisciplinary collaborations continue to thrive, we can envision a future where novel therapies specifically designed to target CSCs contribute to more effective and personalized cancer treatments, ultimately improving patient outcomes and advancing the fight against cancer.

## Author contributions

GA, ML, and JK contributed to the conception, writing, and design of the review. All three performed literature review and drafted separate sections of the manuscript. YL structure, read, revised, and approved the submitted manuscript. All authors contributed to the article and approved the submitted version.

## References

[1] Ferlay J Colombet M Soerjomataram I Parkin DM Piñeros M Znaor A. Cancer statistics for the year 2020: An overview. Int J Cancer (2021) 149(4):778–89. doi: 10.1002/ijc.33588 33818764

[2] Mun JY Leem SH Lee JH Kim HS. Dual relationship between stromal cells and immune cells in the tumor microenvironment. Front Immunol (2022) 13:864739. doi: 10.3389/fimmu.2022.864739 35464435 PMC9019709

[3] Zhang Y Guan XY Jiang P. Cytokine and chemokine signals of T-cell exclusion in tumors. Front Immunol (2020) 11. doi: 10.3389/fimmu.2020.594609 PMC7768018 33381115

[4] Majidpoor J Mortezaee K. Angiogenesis as a hallmark of solid tumors - clinical perspectives. Cell Oncol (Dordr) (2021) 44(4):715–37. doi: 10.1007/s13402-021-00602-3 PMC12980750 33835425

[5] Brassart-Pasco S Brézillon S Brassart B Ramont L Oudart JB Monboisse JC. Tumor microenvironment: Extracellular matrix alterations influence tumor progression. Front Oncol (2020) 10:397. doi: 10.3389/fonc.2020.00397 32351878 PMC7174611

[6] Zhang X Powell K Li L. Breast cancer stem cells: Biomarkers, identification and isolation methods, regulating mechanisms, cellular origin, and beyond. Cancers (Basel) (2020) 12(12):1–28. doi: 10.3390/cancers12123765 PMC7765014 33327542

[7] Kim J Zaret KS. Reprogramming of human cancer cells to pluripotency for models of cancer progression. EMBO J (2015) 34(6):739–47. doi: 10.15252/embj.201490736 PMC4369311 25712212

[8] Fatma H Siddique HR. Pluripotency inducing Yamanaka factors: role in stemness and chemoresistance of liver cancer. Expert Rev Anticancer Ther (2021) 21(8):853–64. doi: 10.1080/14737140.2021.1915137 33832395

[9] Colvin H Mori M. Getting to the heart of the matter in cancer: Novel approaches to targeting cancer stem cells. Proc Jpn Acad Ser B Phys Biol Sci (2017) 93(3):146. doi: 10.2183/pjab.93.009 PMC5422580 28302961

[10] Miyagi-Shiohira C Saitoh I Watanabe M Noguchi H. Gene expression in pancreatic cancer-like cells and induced pancreatic stem cells generated by transient overexpression of reprogramming factors. J Clin Med (2021) 10:454. doi: 10.3390/jcm10030454 33504014 PMC7865593

[11] Caballano-Infantes E Díaz I Hitos AB Cahuana GM Martínez-Ruiz A Soria-Juan B. Stemness of human pluripotent cells: Hypoxia-like response induced by low nitric oxide. Antioxidants (2021) 10(9):1408. doi: 10.3390/antiox10091408 34573040 PMC8472328

[12] López-Anguita N Gassaloglu SI Stötzel M Bolondi A Conkar D Typou M. Hypoxia induces an early primitive streak signature, enhancing spontaneous elongation and lineage representation in gastruloids. Development (2022) 149(20). doi: 10.1242/dev.200679 PMC9578691 36102628

[13] Prigione A Rohwer N Hoffmann S Mlody B Drews K Bukowiecki R. HIF1α Modulates cell fate reprogramming through early glycolytic shift and upregulation of PDK1–3 and PKM2. Stem Cells (2014) 32(2):364–76. doi: 10.1002/stem.1552 PMC5730046 24123565

[14] Soni S Padwad YS. HIF-1 in cancer therapy: two decade long story of a transcription factor. Acta Oncol (2017) 56(4):503–15. doi: 10.1080/0284186X.2017.1301680 28358664

[15] Bonnet D Dick JE. Human acute myeloid leukemia is organized as a hierarchy that originates from a primitive hematopoietic cell. Nat Med (1997) 3:7. doi: 10.1038/nm0797-730 9212098

[16] Al-Hajj M Wicha MS Benito-Hernandez A Morrison SJ Clarke MF. Prospective identification of tumorigenic breast cancer cells. Proc Natl Acad Sci U.S.A. (2003) 100(7):3983–8. doi: 10.1073/pnas.0530291100 PMC153034 12629218

[17] Gong L Yan Q Zhang Y Fang X Liu B Guan X. Cancer cell reprogramming: a promising therapy converting Malignancy to benignity. Cancer Commun (2019) 39:1. doi: 10.1186/s40880-019-0393-5 PMC6716904 31464654

[18] Müller M Hermann PC Liebau S Weidgang C Seufferlein T Kleger A. The role of pluripotency factors to drive stemness in gastrointestinal cancer. Stem Cell Res (2016) 16(2):349–57. doi: 10.1016/j.scr.2016.02.005 26896855

[19] Hepburn AC Steele RE Veeratterapillay R Wilson L Kounatidou EE Barnard A. The induction of core pluripotency master regulators in cancers defines poor clinical outcomes and treatment resistance. Oncogene (2019) 38:22. doi: 10.1038/s41388-019-0712-y PMC6546609 30742096

[20] Sell S. On the stem cell origin of cancer. Am J Pathol (2010) 176(6):2584. doi: 10.2353/ajpath.2010.091064 20431026 PMC2877820

[21] Sell S. Maturation arrest of stem cell differentiation is a common pathway for the cellular origin of teratocarcinomas and epithelial cancers. Lab Invest (2022) 70(1):6–22. 8302019

[22] Lapidot T Sirard C Vormoor J Murdoch B Nature TH. A cell initiating human acute myeloid leukaemia after transplantation into SCID mice. Nature (2022) 367:645–8. doi: 10.1038/367645a0 7509044

[23] Sutherland H Blair A Zapf R. Characterization of a hierarchy in human acute myeloid leukemia progenitor cells. Blood (1996) 11:4754–61. doi: 10.1182/blood.V87.11.4754.bloodjournal87114754 8639846

[24] López J Valdez-Morales FJ Benítez-Bribiesca L Cerbón M Carrancá AG. Normal and cancer stem cells of the human female reproductive system. Reprod Biol Endocrinol (2013) 11(1):53. doi: 10.1186/1477-7827-11-53 23782518 PMC3693871

[25] Ayob AZ Ramasamy TS. Cancer stem cells as key drivers of tumour progression. J Biomed Sci (2018) 25:1. doi: 10.1186/s12929-018-0426-4 29506506 PMC5838954

[26] Carvalho J. Cell reversal from a differentiated to a stem-like state at cancer initiation. Front Oncol (2020) 10:541. doi: 10.3389/fonc.2020.00541 32351900 PMC7174973

[27] Wang RA Li ZS Zhang HZ Zheng PJ Li QL Shi JG. Invasive cancers are not necessarily from preformed in situ tumours — an alternative way of carcinogenesis from misplaced stem cells. J Cell Mol Med (2013) 17(7):921. doi: 10.1111/jcmm.12078 23741988 PMC3822897

[28] Walcher L Kistenmacher AK Suo H Kitte R Dluczek S Strauß A. Cancer stem cells—Origins and biomarkers: Perspectives for targeted personalized therapies. Front Immunol (2020) 11:1280. doi: 10.3389/fimmu.2020.01280 32849491 PMC7426526

[29] Afify SM Seno M. Conversion of stem cells to cancer stem cells: Undercurrent of cancer initiation. Cancers (Basel) (2019) 11(3):345. doi: 10.3390/cancers11030345 30862050 PMC6468812

[30] Ratajczak MZ Bujko K Mack A Kucia M Ratajczak J. Cancer from the perspective of stem cells and misappropriated tissue regeneration mechanisms. Leukemia (2018) 32:12. doi: 10.1038/s41375-018-0294-7 PMC6286324 30375490

[31] Emanuele Liardo RL Borzì AM Spatola C Martino B Privitera G Basile F. Effects of infections on the pathogenesis of cancer. Indian J Med Res (2021) 153(4):431. doi: 10.4103/ijmr.IJMR_339_19 34380789 PMC8354054

[32] Cohen L Jefferies A. Environmental exposures and cancer: using the precautionary principle. Ecancermedicalscience (2019) 13(13):ed91. doi: 10.3332/ecancer.2019.ed91 31281435 PMC6546253

[33] Wu S Zhu W Thompson P Hannun YA. Evaluating intrinsic and non-intrinsic cancer risk factors. Nat Commun (2018) 9:1. doi: 10.1038/s41467-018-05467-z 30154431 PMC6113228

[34] Li Z Zhang YY Zhang H Yang J Chen Y Lu H. Asymmetric cell division and tumor heterogeneity. Front Cell Dev Biol (2022) 10:1374. doi: 10.3389/fcell.2022.938685 PMC9289117 35859890

[35] Kaushik V Kulkarni Y Felix K Azad N v. IAK Yakisich JS. Alternative models of cancer stem cells: The stemness phenotype model, 10 years later. World J Stem Cells (2021) 13(7):934–43. doi: 10.4252/wjsc.v13.i7.934 PMC8316871 34367485

[36] Venizelos A Engebrethsen C Deng W Geisler J Geisler S Iversen GT. Clonal evolution in primary breast cancers under sequential epirubicin and docetaxel monotherapy. Genome Med (2022) 14(1):1–18. doi: 10.1186/s13073-022-01090-2 35948919 PMC9367103

[37] Thankamony AP Saxena K Murali R Jolly MK Nair R. Cancer stem cell plasticity – A deadly deal. Front Mol Biosci (2020) 7:79. doi: 10.3389/fmolb.2020.00079 32426371 PMC7203492

[38] Bhatia M Wang JCY Kapp U Bonnet D Dick JE. Purification of primitive human hematopoietic cells capable of repopulating immune-deficient mice. Proc Natl Acad Sci USA (1997) 94(10):5320–5. doi: 10.1073/pnas.94.10.5320 PMC24676 9144235

[39] Hansen Q Bachas C Smit L Cloos J. Characteristics of leukemic stem cells in acute leukemia and potential targeted therapies for their specific eradication. Cancer Drug Resistance (2022) 5(2):344. doi: 10.20517/cdr.2021.140 35800375 PMC9255252

[40] Issah MA Wu D Zhang F Zheng W Liu Y Fu H. Epigenetic modifications in acute myeloid leukemia: The emerging role of circular RNAs (Review). Int J Oncol (2021) 59(6):107. doi: 10.3892/ijo.2021.5287 34792180 PMC8651224

[41] Jan M Snyder TM Corces-Zimmerman MR Vyas P Weissman IL Quake SR. Clonal evolution of preleukemic hematopoietic stem cells precedes human acute myeloid leukemia. Sci Transl Med (2012) 4(149):149ra118. doi: 10.1126/scitranslmed.3004315 PMC4045621 22932223

[42] Bray F Ferlay J Soerjomataram I Siegel RL Torre LA Jemal A. Global cancer statistics 2018: GLOBOCAN estimates of incidence and mortality worldwide for 36 cancers in 185 countries. CA Cancer J Clin (2018) 68(6):394–424. doi: 10.3322/caac.21492 30207593

[43] Vaupel P Mayer A. Hypoxia in cancer: significance and impact on clinical outcome. Cancer Metastasis Rev (2007) 26(2):225–39. doi: 10.1007/s10555-007-9055-1 17440684

[44] Bharti SK Kakkad S Danhier P Wildes F Penet MF Krishnamachary B. Hypoxia patterns in primary and metastatic prostate cancer environments. Neoplasia (2019) 21(2):239. doi: 10.1016/j.neo.2018.12.004 30639975 PMC6327878

[45] Lyng H Malinen E. Hypoxia in cervical cancer: from biology to imaging. Clin Transl Imaging (2017) 5(4):373. doi: 10.1007/s40336-017-0238-7 28804704 PMC5532411

[46] Liu Q Palmgren VAC Danen EH Le Dévédec SE. Acute vs. chronic vs. intermittent hypoxia in breast Cancer: a review on its application in in vitro research. Mol Biol Rep (2022) 49:11. doi: 10.1007/s11033-022-07802-6 PMC9618509 36057753

[47] Wegge M Dok R Nuyts S. Hypoxia and its influence on radiotherapy response of HPV-positive and HPV-negative head and neck cancer. Cancers (2021) 13:5959. doi: 10.3390/cancers13235959 34885069 PMC8656584

[48] Vaupel P Flood AB Swartz HM. Oxygenation Status of Malignant Tumors vs. Normal Tissues: Critical Evaluation and Updated Data Source Based on Direct Measurements with pO2 Microsensors. Appl Magnetic Resonance (2021) 52:10. doi: 10.1007/s00723-021-01383-6

[49] Yan Y Liu F Han L Zhao L Chen J Olopade OI. HIF-2α promotes conversion to a stem cell phenotype and induces chemoresistance in breast cancer cells by activating Wnt and Notch pathways. J Exp Clin Cancer Res (2018) 37(1):1–14. doi: 10.1186/s13046-018-0925-x 30340507 PMC6194720

[50] Zhang Q Han Z Zhu Y Chen J Li W. Role of hypoxia inducible factor-1 in cancer stem cells. Mol Med Rep (2021) 23(1):17. doi: 10.3892/mmr.2020.11655 33179080 PMC7673349

[51] Kolonko M Greb-Markiewicz B. bHLH–PAS proteins: Their structure and intrinsic disorder. Int J Mol Sci (2019) 20(15):3653. doi: 10.3390/ijms20153653 31357385 PMC6695611

[52] Peng G Liu Y. Hypoxia-inducible factors in cancer stem cells and inflammation. Trends Pharmacol Sci (2015) 36(6):374. doi: 10.1016/j.tips.2015.03.003 25857287 PMC4461458

[53] Zhang P Yao Q Lu L Li Y Chen PJ Duan C. Hypoxia-inducible factor 3 is an oxygen-dependent transcription activator and regulates a distinct transcriptional response to hypoxia. Cell Rep (2014) 6(6):1110–21. doi: 10.1016/j.celrep.2014.02.011 24613356

[54] Smythies JA Sun M Masson N Salama R Simpson PD Murray E. Inherent DNA-binding specificities of the HIF-1α and HIF-2α transcription factors in chromatin. EMBO Rep (2019) 20(1):e46401. doi: 10.15252/embr.201846401 30429208 PMC6322389

[55] Li Z Bao S Wu Q Wang H Eyler C Sathornsumetee S. Hypoxia-inducible factors regulate tumorigenic capacity of glioma stem cells. Cancer Cell (2009) 15(6):501–13. doi: 10.1016/j.ccr.2009.03.018 PMC2693960 19477429

[56] Zhao L Liu Z Yang F Zhang Y Xue Y Miao H. Intrabody against prolyl hydroxylase 2 promotes angiogenesis by stabilizing hypoxia-inducible factor-1α. Sci Rep (2019) 9:1. doi: 10.1038/s41598-019-47891-1 31413262 PMC6694103

[57] Nagao A Kobayashi M Koyasu S Chow CCT Harada H. HIF-1-dependent reprogramming of glucose metabolic pathway of cancer cells and its therapeutic significance. Int J Mol Sci (2019) 20:238. doi: 10.3390/ijms20020238 30634433 PMC6359724

[58] Jin X Dai L Ma Y Wang J Liu Z. Implications of HIF-1α in the tumorigenesis and progression of pancreatic cancer. Cancer Cell Int (2020) 20:1. doi: 10.1186/s12935-020-01370-0 32587480 PMC7313137

[59] Tam SY Wu VWC Law HKW. Hypoxia-induced epithelial-mesenchymal transition in cancers: HIF-1α and beyond. Front Oncol (2020) 10:486. doi: 10.3389/fonc.2020.00486 32322559 PMC7156534

[60] Wang P Gong S Liao B Pan J Wang J Zou D. HIF1α/HIF2α induces glioma cell dedifferentiation into cancer stem cells through Sox2 under hypoxic conditions. J Cancer (2022) 13(1):1. doi: 10.7150/jca.54402 34976166 PMC8692689

[61] Kwak JH Lee NH Lee HY Hong IS Nam JS Kwak JH. HIF2α/EFEMP1 cascade mediates hypoxic effects on breast cancer stem cell hierarchy. Oncotarget (2016) 7(28):43518–33. doi: 10.18632/oncotarget.9846 PMC5190041 27270657

[62] Takahashi K Yamanaka S. Induction of pluripotent stem cells from mouse embryonic and adult fibroblast cultures by defined factors. Cell (2006) 126(4):663–76. doi: 10.1016/j.cell.2006.07.024 16904174

[63] Zhao W Li Y Zhang X. Stemness-related markers in cancer. Cancer Transl Med (2017) 3(3):87. doi: 10.4103/ctm.ctm_69_16 29276782 PMC5737740

[64] Nakamura N Shi X Darabi R Li Y. Hypoxia in cell reprogramming and the epigenetic regulations. Front Cell Dev Biol (2021) 9. doi: 10.3389/fcell.2021.609984 PMC7876330 33585477

[65] Bernhardt M Novak D Assenov Y Orouji E Knappe N Weina K. Melanoma-derived iPCCs show differential tumorigenicity and therapy response. Stem Cell Rep (2017) 8(5):1379. doi: 10.1016/j.stemcr.2017.03.007 PMC5425615 28392221

[66] Zhang S Xiong X Sun Y. Functional characterization of SOX2 as an anticancer target. Signal Transduction Targeted Ther (2020) 5:1. doi: 10.1038/s41392-020-00242-3 PMC7391717 32728033

[67] Feng R Wen J. Overview of the roles of Sox2 in stem cell and development. Biol Chem (2015) 396(8):883–91. doi: 10.1515/hsz-2014-0317 25781683

[68] Wang Y Bibi M Min P Deng W Zhang Y Du J. SOX2 promotes hypoxia-induced breast cancer cell migration by inducing NEDD9 expression and subsequent activation of Rac1/HIF-1α signaling. Cell Mol Biol Lett (2019) 24(1):55. doi: 10.14715/cmb/2019.65.7.2 31462898 PMC6704701

[69] Mu P Zhang Z Benelli M Karthaus WR Hoover E Chen CC. SOX2 promotes lineage plasticity and antiandrogen resistance in TP53- and RB1-deficient prostate cancer. Science (2017) 355(6320):84–8. doi: 10.1126/science.aah4307 PMC5247742 28059768

[70] Liu P Tang H Song C Wang J Chen B Huang X. SOX2 promotes cell proliferation and metastasis in triple negative breast cancer. Front Pharmacol (2018) 9(AUG). doi: 10.3389/fphar.2018.00942 PMC6110877 30186173

[71] Xu Y Dong X Qi P Ye Y Shen W Leng L. Sox2 communicates with tregs through CCL1 to promote the stemness property of breast cancer cells. Stem Cells (2017) 35(12):2351–65. doi: 10.1002/stem.2720 PMC5958902 29044882

[72] Niu T Zhang W Xiao W. MicroRNA regulation of cancer stem cells in the pathogenesis of breast cancer. Cancer Cell Int (2021) 21(1):31. doi: 10.1186/s12935-020-01716-8 33413418 PMC7792222

[73] Zhang J Cheng Q Zhou Y Wang Y Chen X. Slug is a key mediator of hypoxia induced cadherin switch in HNSCC: Correlations with poor prognosis. Oral Oncol (2013) 49(11):1043–50. doi: 10.1016/j.oraloncology.2013.08.003 24035721

[74] Gao H Teng C Huang W Peng J Wang C. SOX2 promotes the epithelial to mesenchymal transition of esophageal squamous cells by modulating slug expression through the activation of STAT3/HIF-α Signaling. Int J Mol Sci (2015) 16(9):21643–57. doi: 10.3390/ijms160921643 PMC4613272 26370982

[75] Bae KM Dai Y Vieweg J Siemann DW. Hypoxia regulates SOX2 expression to promote prostate cancer cell invasion and sphere formation. Am J Cancer Res (2016) 6(5):1078–88. PMC4889721 27294000

[76] Lu X Mazur SJ Lin T Appella E Xu Y. The pluripotency factor nanog promotes breast cancer tumorigenesis and metastasis. Oncogene (2014) 33:20. doi: 10.1038/onc.2013.209 PMC3925756 23770853

[77] Cheng W Wang H Yuan J Cheng Z Xing D Zhang M. The prognostic value of nanog overexpression in lung cancer: A meta-analysis. BioMed Res Int (2018) 2018. doi: 10.1155/2018/3429261 PMC6304555 30627549

[78] Lin T Ding YQ Li JM. Overexpression of Nanog protein is associated with poor prognosis in gastric adenocarcinoma. Med Oncol (2012) 29(2):878–85. doi: 10.1007/s12032-011-9860-9 21336986

[79] Yang L Shi P Zhao G Xu J Peng W Zhang J. Targeting cancer stem cell pathways for cancer therapy. Signal Transduct Target Ther (2020) 5(1):8. doi: 10.1038/s41392-020-0110-5 32296030 PMC7005297

[80] Jung GA Kim JA Park HW Lee H Chang MS Cho KO. Induction of Nanog in neural progenitor cells for adaptive regeneration of ischemic brain. Exp Mol Med (2022) 54(11):1955–66. doi: 10.1038/s12276-022-00880-3 PMC9722910 36376495

[81] Lu Y Zhu H Shan H Lu J Chang X Li X. Knockdown of Oct4 and Nanog expression inhibits the stemness of pancreatic cancer cells. Cancer Lett (2013) 340(1):113–23. doi: 10.1016/j.canlet.2013.07.009 23872274

[82] Hasmim M Janji B Khaled M NOman MZ Louache F Bordereaux D. Cutting edge: NANOG activates autophagy under hypoxic stress by binding to BNIP3L promoter. J Immunol (2017) 198(4):1423–8. doi: 10.4049/jimmunol.1600981 28093523

[83] Ibrahim EE Babaei-Jadidi R Saadeddin A Spencer-Dene B Hossaini S Abuzinadah M. Embryonic NANOG activity defines colorectal cancer stem cells and modulates through AP1- and TCF-dependent mechanisms. Stem Cells (2012) 30(10):2076–87. doi: 10.1002/stem.1182 22851508

[84] Hasmim M NOman MZ Lauriol J Benlalam H Mallavialle A Rosselli F. Hypoxia-dependent inhibition of tumor cell susceptibility to CTL-mediated lysis involves NANOG induction in target cells. J Immunol (2011) 187(8):4031–9. doi: 10.4049/jimmunol.1101011 21911602

[85] Lu H Lyu Y Tran L Lan J Xie Y Yang Y. HIF-1 recruits NANOG as a coactivator for TERT gene transcription in hypoxic breast cancer stem cells. Cell Rep (2021) 36(13):109757. doi: 10.1016/j.celrep.2021.109757 34592152

[86] Zhang C Samanta D Lu H Bullen JW Zhang H Chen I. Hypoxia induces the breast cancer stem cell phenotype by HIF-dependent and ALKBH5-mediated m ^6^ A-demethylation of NANOG mRNA. Proc Natl Acad Sci (2016) 113(14):E2047–56. doi: 10.1073/pnas.1602883113 PMC4833258 27001847

[87] Hasmim M NOman MZ Messai Y Bordereaux D Gros G Baud V. Cutting edge: Hypoxia-induced nanog favors the intratumoral infiltration of regulatory T cells and macrophages *via* direct regulation of TGF-β1. J Immunol (2013) 191(12):5802–6. doi: 10.4049/jimmunol.1302140 24227785

[88] Liu Y Yang M Luo J Zhou H. Radiotherapy targeting cancer stem cells “awakens” them to induce tumour relapse and metastasis in oral cancer. Int J Oral Sci (2020) 12(1):19. doi: 10.1038/s41368-020-00087-0 32576817 PMC7311531

[89] Cho Y Kang HG Kim SJ Lee S Jee S Ahn SG. Post-translational modification of OCT4 in breast cancer tumorigenesis. Cell Death Differ (2018) 25(10):1781–95. doi: 10.1038/s41418-018-0079-6 PMC6180041 29511337

[90] Hatefi N Nouraee N Parvin M Ziaee SAM Mowla SJ. Evaluating the expression of oct4 as a prognostic tumor marker in bladder cancer. Iran J Basic Med Sci (2012) 15(6):1154. 23653844 PMC3646225

[91] Yin X Zhang BH Zheng SS Gao DM Qiu SJ Wu WZ. Coexpression of gene Oct4 and Nanog initiates stem cell characteristics in hepatocellular carcinoma and promotes epithelial-mesenchymal transition through activation of Stat3/Snail signaling. J Hematol Oncol (2015) 8(1):23. doi: 10.1186/s13045-015-0119-3 25879771 PMC4377043

[92] Song B Kim DK Shin J Bae SH Kim HY Won B. OCT4 directly regulates stemness and extracellular matrix-related genes in human germ cell tumours. Biochem Biophys Res Commun (2018) 503(3):1980–6. doi: 10.1016/j.bbrc.2018.07.145 30078675

[93] Xiong S Wang D Tang Y Lu S Huang L Wu Z. HIF1α and HIF2α regulate non-small-cell lung cancer dedifferentiation *via* expression of Sox2 and Oct4 under hypoxic conditions. Gene. (2023) 863:147288. doi: 10.1016/j.gene.2023.147288 36804853

[94] Lin SC Chung CH Chung CH Kuo MH Hsieh CH Chiu YF. OCT4B mediates hypoxia-induced cancer dissemination. Oncogene (2019) 38(7):1093–105. doi: 10.1038/s41388-018-0487-6 30209362

[95] Liu HL ting TH lin YH TT D YP Xu SQ Xu. Oct4 regulates the transition of cancer stem-like cells to tumor endothelial-like cells in human liver cancer. Front Cell Dev Biol (2020) 8. doi: 10.3389/fcell.2020.563316 PMC7554317 33102474

[96] Wang Y Yang C Gu Q Sims M Gu W Pfeffer LM. KLF4 promotes angiogenesis by activating VEGF signaling in human retinal microvascular endothelial cells. PLoS One (2015) 10(6):e0130341. doi: 10.1371/journal.pone.0130341 26075898 PMC4467843

[97] Riverso M Montagnani V Stecca B. KLF4 is regulated by RAS/RAF/MEK/ERK signaling through E2F1 and promotes melanoma cell growth. Oncogene (2017) 36(23):3322–33. doi: 10.1038/onc.2016.481 PMC5474568 28068326

[98] Wei D Kanai M Huang S Xie K. Emerging role of KLF4 in human gastrointestinal cancer. Carcinogenesis. (2005) 27(1):23–31. doi: 10.1093/carcin/bgi243 16219632

[99] Wang P Zhao L Gong S Xiong S Wang J Zou D. HIF1α/HIF2α–Sox2/Klf4 promotes the Malignant progression of glioblastoma via the EGFR–PI3K/AKT signalling pathway with positive feedback under hypoxia. Cell Death Dis (2021) 12(4):312. doi: 10.1038/s41419-021-03598-8 33762574 PMC7990922

[100] Shan F Huang Z Xiong R Huang QY Li J. HIF1α-induced upregulation of KLF4 promotes migration of human vascular smooth muscle cells under hypoxia. J Cell Physiol (2020) 235(1):141–50. doi: 10.1002/jcp.28953 31270801

[101] Zhang Y Wang S Hu H Li X. A systematic study of HIF1A cofactors in hypoxic cancer cells. Sci Rep (2022) 12(1):18962. doi: 10.1038/s41598-022-23060-9 36347941 PMC9643333

[102] Wang T Guo Y Liu S Zhang C Cui T Ding K. KLF4, a key regulator of a transitive triplet, acts on the TGF-β Signaling pathway and contributes to high-altitude adaptation of tibetan pigs. Front Genet (2021) 12. doi: 10.3389/fgene.2021.628192 PMC8082500 33936161

[103] Kawanami D Mahabeleshwar GH Lin Z Atkins GB Hamik A Haldar SM. Kruppel-like factor 2 inhibits hypoxia-inducible factor 1α Expression and function in the endothelium. J Biol Chem (2009) 284(31):20522–30. doi: 10.1074/jbc.M109.025346 PMC2742816 19491109

[104] Poli V Fagnocchi L Fasciani A Cherubini A Mazzoleni S Ferrillo S. MYC-driven epigenetic reprogramming favors the onset of tumorigenesis by inducing a stem cell-like state. Nat Commun (2018) 9(1):1024. doi: 10.1038/s41467-018-03264-2 29523784 PMC5844884

[105] Li Y Sun XX Qian DZ Dai MS. Molecular crosstalk between MYC and HIF in cancer. Front Cell Dev Biol (2020) 8. doi: 10.3389/fcell.2020.590576 PMC7676913 33251216

[106] Zhu P He F Hou Y Tu G Li Q Jin T. A novel hypoxic long noncoding RNA KB-1980E6.3 maintains breast cancer stem cell stemness *via* interacting with IGF2BP1 to facilitate c-Myc mRNA stability. Oncogene (2021) 40(9):1609–27. doi: 10.1038/s41388-020-01638-9 PMC7932928 33469161

[107] Greaves M Maley CC. Clonal evolution in cancer. Nature (2012) 481:306–13. doi: 10.1038/nature10762 PMC3367003 22258609

[108] Lambert AW Pattabiraman DR Weinberg RA. Emerging biological principles of metastasis. Cell (2017) 168:670–91. doi: 10.1016/j.cell.2016.11.037 PMC5308465 28187288

[109] Chang JC. Cancer stem cells: Role in tumor growth, recurrence, metastasis, and treatment resistance. Med (United States) (2016) 95:S20–5. doi: 10.1097/MD.0000000000004766 PMC5599212 27611935

[110] Lai X Li Q Wu F Lin J Chen J Zheng H. Epithelial-mesenchymal transition and metabolic switching in cancer: lessons from somatic cell reprogramming. Front Cell Dev Biol (2020) 8. doi: 10.3389/fcell.2020.00760 PMC7423833 32850862

[111] Rossi F Noren H Jove R Beljanski V Grinnemo KH. Differences and similarities between cancer and somatic stem cells: therapeutic implications. Stem Cell Res Ther (2020) 11:489. doi: 10.1186/s13287-020-02018-6 33208173 PMC7672862

[112] Yang J Weinberg RA. Epithelial-mesenchymal transition: At the crossroads of development and tumor metastasis. Dev Cell (2008) 14:818–29. doi: 10.1016/j.devcel.2008.05.009 18539112

[113] Brabletz T Jung A Spaderna S Hlubek F Kirchner T. Migrating cancer stem cells-an integrated concept of Malignant tumour progression. Nat Rev Cancer (2005) 5:744–9. doi: 10.1038/nrc1694 16148886

[114] Muz B de la Puente P Azab F Azab AK. The role of hypoxia in cancer progression, angiogenesis, metastasis, and resistance to therapy. Hypoxia (2015) 83:83–92. doi: 10.2147/HP.S93413 PMC5045092 27774485

[115] Saito S Lin YC Nakamura Y Eckner R Wuputra K Kuo KK. Potential application of cell reprogramming techniques for cancer research. Cell Mol Life Sci (2019) 76:45–65. doi: 10.1007/s00018-018-2924-7 30283976 PMC6326983

[116] Zhao D Pan C Sun J Gilbert C Drews-Elger K Azzam DJ. VEGF drives cancer-initiating stem cells through VEGFR-2/Stat3 signaling to upregulate Myc and Sox2. Oncogene (2015) 34(24):3107–19. doi: 10.1038/onc.2014.257 25151964

[117] Hillen F Griffioen AW. Tumour vascularization: Sprouting angiogenesis and beyond. Cancer Metastasis Rev (2007) 26:489–502. doi: 10.1007/s10555-007-9094-7 17717633 PMC2797856

[118] Maniotis AJ Folberg R Hess A Seftor EA Gardner LM Pe'er J. Vascular channel formation by human melanoma cells in Vivo and in vitro: Vasculogenic mimicry. Am J Pathol (1999) 155. doi: 10.1016/S0002-9440(10)65173-5 PMC1866899 10487832

[119] Lin P Wang W Sun Bc Cai Wj Li L Lu Hh. Vasculogenic mimicry is a key prognostic factor for laryngeal squamous cell carcinoma: A new pattern of blood supply. Chin Med J (2012) 125:3445–9. 23044304

[120] Sun H Yao N Cheng S Li L Liu S Yang Z. Cancer stem-like cells directly participate in vasculogenic mimicry channels in triple-negative breast cancer. Cancer Biol Med (2019) 16(2):299–311. 31516750 10.20892/j.issn.2095-3941.2018.0209 PMC6713644

[121] Zhang D Sun B Zhao X Ma Y Ji R Gu Q. Twist1 expression induced by sunitinib accelerates tumor cell vasculogenic mimicry by increasing the population of CD133+ cells in triple-negative breast cancer. Mol Cancer (2014) 13(1). doi: 10.1186/1476-4598-13-207 PMC4168051 25200065

[122] Siegel RL Miller KD Wagle NS Jemal A. Cancer statistics, 2023. CA Cancer J Clin (2023) 73(1):17–48. doi: 10.3322/caac.21763 36633525

[123] Debela DT Muzazu SG Heraro KD Ndalama MT Mesele BW Haile DC. New approaches and procedures for cancer treatment: Current perspectives. SAGE Open Med (2021) 9:205031212110343. doi: 10.1177/20503121211034366 PMC8366192 34408877

[124] Codony VL Tavassoli M. Hypoxia-induced therapy resistance: Available hypoxia-targeting strategies and current advances in head and neck cancer. Transl Oncol (2021) 14(3):101017. doi: 10.1016/j.tranon.2021.101017 33465746 PMC7814189

[125] Schulz A Meyer F Dubrovska A Borgmann K. Cancer stem cells and radioresistance: DNA repair and beyond. Cancers (Basel) (2019) 11(6):862. doi: 10.3390/cancers11060862 31234336 PMC6627210

[126] Rockwell S Dobrucki I Kim E Marrison S Vu V. Hypoxia and radiation therapy: Past history, ongoing research, and future promise. Curr Mol Med (2009) 9(4):442–58. doi: 10.2174/156652409788167087 PMC2752413 19519402

[127] Zhu XH Du JX Zhu D Ren SZ Chen K Zhu HL. Recent research on methods to improve tumor hypoxia environment. Oxid Med Cell Longev (2020) 2020:1–18. doi: 10.1155/2020/5721258 PMC7725563 33343807

[128] Gaggianesi M Di Franco S Pantina VD Porcelli G D’Accardo C Verona F. Messing up the cancer stem cell chemoresistance mechanisms supported by tumor microenvironment. Front Oncol (2021) 11. doi: 10.3389/fonc.2021.702642 PMC8330815 34354950

[129] Röhrig F Vorlová S Hoffmann H Wartenberg M Escorcia FE Keller S. VEGF-ablation therapy reduces drug delivery and therapeutic response in ECM-dense tumors. Oncogene (2017) 36:1. doi: 10.1038/onc.2016.182 27270432 PMC5237662

[130] Bondhopadhyay B Sisodiya S Chikara A Khan A Tanwar P Afroze D. Cancer immunotherapy: a promising dawn in cancer research. Am J Blood Res (2020) 10(6):375. 33489447 PMC7811907

[131] He Z Zhang S. Tumor-associated macrophages and their functional transformation in the hypoxic tumor microenvironment. Front Immunol (2021) 12:3819. doi: 10.3389/fimmu.2021.741305 PMC8481680 34603327

[132] Iranmanesh Y Jiang B Favour OC Dou Z Wu J Li J. Mitochondria’s role in the maintenance of cancer stem cells in glioblastoma. Front Oncol (2021) 11:101. doi: 10.3389/fonc.2021.582694 PMC7937970 33692947

[133] Sun HR Wang S Yan SC Zhang Y Nelson PJ Jia HL. Therapeutic strategies targeting cancer stem cells and their microenvironment. Front Oncol (2019) 9(OCT):1104. doi: 10.3389/fonc.2019.01104 31709180 PMC6821685

[134] Ghasemi S Xu S Nabavi SM Amirkhani MA Sureda A Tejada S. Epigenetic targeting of cancer stem cells by polyphenols (cancer stem cells targeting). Phytotherapy Res (2021) 35(7):3649–64. doi: 10.1002/ptr.7059 33619811

[135] Peng S Maihle NJ Huang Y. Pluripotency factors Lin28 and Oct4 identify a sub-population of stem cell-like cells in ovarian cancer. Oncogene (2010) 29:14. doi: 10.1038/onc.2009.500 20101213

[136] Lu CS Hsieh JL Lin CY Tsai HW Su BH Shieh GS. Potent antitumor activity of Oct4 and hypoxia dual-regulated oncolytic adenovirus against bladder cancer. Gene Ther (2015) 22(4):305–15. doi: 10.1038/gt.2014.122 25588741

[137] Zimmermannova O Caiado I Ferreira AG Pereira CF. Cell fate reprogramming in the era of cancer immunotherapy. Front Immunol (2021) 12:2934. doi: 10.3389/fimmu.2021.714822 PMC8336566 34367185

[138] Linde MH Fan AC Kohnke T Trotman-Grant AC Gurev SF Phan P. Reprogramming cancer into antigen presenting cells as a novel immunotherapy. Cancer Discovery (2023) 13(5):1164–85. doi: 10.1158/2159-8290.CD-21-0502/716614/Reprogramming-Cancer-into-Antigen-Presenting-Cells 36856575

[139] Suzuka J Tsuda M Wang L Kohsaka S Kishida K Semba S. Rapid reprogramming of tumour cells into cancer stem cells on double-network hydrogels. Nat Biomed Eng (2021) 5:8. doi: 10.1038/s41551-021-00692-2 33782572

[140] Bang JS Choi NY Lee M Ko K Park YS Ko K. Reprogramming of cancer cells into induced pluripotent stem cells questioned. Int J Stem Cells (2019) 12(3):430. doi: 10.15283/ijsc19067 31474029 PMC6881048

[141] Izgi K Canatan H Iskender B. Current status in cancer cell reprogramming and its clinical implications. J Cancer Res Clin Oncol (2017) 143(3):371–83. doi: 10.1007/s00432-016-2258-5 PMC11819102 27620745

